# 2018 FDA Tides Harvest

**DOI:** 10.3390/ph12020052

**Published:** 2019-04-05

**Authors:** Danah Al Shaer, Othman Al Musaimi, Fernando Albericio, Beatriz G. de la Torre

**Affiliations:** 1 KRISP, School of Laboratory of Medicine and Medical Science, College of Health Sciences, University of KwaZulu-Natal, Durban 4001, South Africa; 217078895@stuukznac.onmicrosoft.com (D.A.S.); 217078894@stuukznac.onmicrosoft.com (O.A.M.); 2 School of Chemistry and Physics, University of KwaZulu-Natal, Durban 4001, South Africa; 3 CIBER-BBN, Networking Centre on Bioengineering, Biomaterials and Nanomedicine and Department of Organic Chemistry, University of Barcelona, 08028 Barcelona, Spain

**Keywords:** dotatate, drugs, inotersen, Lutathera, oligonucleotides, Onpattro, patisiran, peptides, pharmaceutical market, Tegsedi

## Abstract

In 2018, the United States Food and Drug Administration (FDA) approved a total of 59 new drugs, three of them (5%) are TIDES (or also, -tides), two oligonucleotides and one peptide. Herein, the three TIDES approved are analyzed in terms of medical target, mode of action, chemical structure, and economics.

## 1. Introduction

Drug discovery is a unique transdisciplinary activity involving a wide range of interconnected parameters, thus posing a challenge for the pharmaceutical industry and related fields. The mission of this industry is to improve or restore health and wellbeing. Indeed, the administration of an appropriate treatment can very often save lives. Although the pharmaceutical industry was devoted mainly to research and development after the second world war, over the years economics has increasingly gained relevance. Between 12 and 20 years can lapse from the time a discovery has been made in a research laboratory and a product reaching the market, and this development pipeline carries with it an associated cost of up to US $ 1–2 billion. What are the main reasons behind these long development times and high costs? There is probably no single answer. However, the main causes can be attributed to the increasingly stricter requirements of the corresponding agencies, mainly the federal Food and Drug Administration (FDA) in the USA, and the European Medicines Agency (EMA) in Europe, regarding toxicity and safety profiles, and the need for new drugs to show superior performance to that of those already on the market.

**Figure 1:**
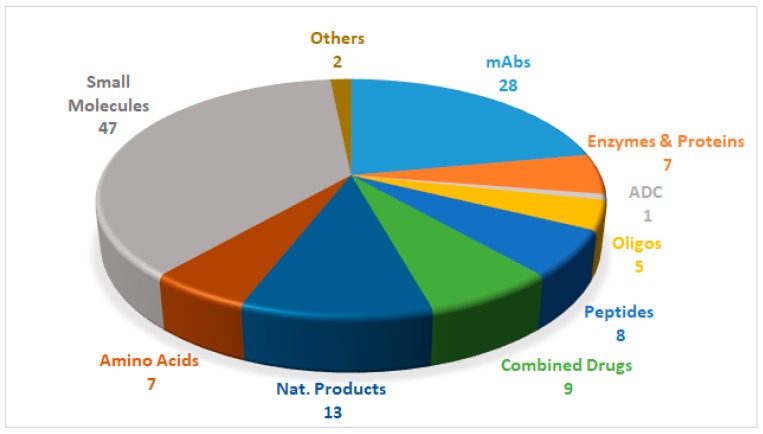
127 new drugs approved by the United States Federal Food and Drug Administration (FDA) from 2016 to 2018, and classified on the basis of chemical structure [2,4,6].

Drugs can be roughly divided into two main categories, namely biologics and chemical entities, the former being prepared by means of biotechnological techniques, and the latter by chemical synthesis. Figure 1 shows the 127 new drugs approved by the FDA in the last three years (2016–2018) (59 in 2018), classified on the basis of chemical structure [1,2,3,4,5,6]. In this period, 36 biologics (17 in 2018) and 91 chemical entities (42 in 2018) were endorsed by the FDA. In the biologics field, the predominance of monoclonal antibodies (mAbs) is remarkable (28 in 2016–2018, and 12 in just the last year). As chemical entities, the so-called small molecules continue to account for a considerable proportion of new drugs. Natural product-based drugs and TIDES (oligonucleo- and pep-TIDES) each represent approximately 10% of new drugs [7]. Indeed, there are currently 14 drugs (10 in 2018) inspired in natural products, and 13 TIDES (three in 2018) on the market. 

Although only one peptide received FDA approval in 2018—a significant drop from the previous year in which six were approved—2018 has been extremely important for oligonucleotides, with two approvals. In support of this statement, only six oligonucleotide drugs have received FDA approval to date (three in 2016).

Herein, the three TIDES (one peptide and two oligonucleotides) with FDA approval are analyzed in terms of therapeutic use, mode of action, and chemical structures.

## 2. Oligonucleotides

The two oligonucleotides in the 2018 harvest, patisiran (Onpattro^TM^) and inotersen (Tegsedi^TM^), are prescribed for the treatment of polyneuropathy hereditary transthyretin mediated amyloidosis (hATTR) in adults.

Transthyretin (TTR or TBPA) is a transport protein that carries the thyroid hormone, thyroxine (T4), and the retinol-binding protein when it is bound to retinol (vitamin A) [8]. The liver is the main secretor of TTR into the bloodstream, and the choroid plexus secretes it into the cerebrospinal fluid. TTR is formed by four monomers of 127 amino acids, rich in β-sheet structures. Its final quaternary structure is a result of the association of two dimers in a face to face arrangement, creating a central channel that is responsible for two binding sites [8].

Individuals with hATTR show a mutation in the TTR gene, which in the protein is translated by replacing Val for Met at position 30 [9]. The mutation leads to an altered TTR protein structure [10], thus causing more dissociation at the dimer-dimer interface than in the wild-type. This dissociation results in the accumulation of the misfolded protein as insoluble amyloid fibrils in multiple organs, including nerves, heart, and gastrointestinal tract [11], and ultimately causes the disorder.

Previous treatments for this disease involved orthotopic liver transplantation (OLT) [10] and the use of some drugs, such as tafamidis [12] and diflunisal [13]. These act as TTR protein stabilizers, and thus delay the deposition of fibrils.

### 2.1. Patisiran (Onpattro^TM^)

The approval of patisiran (Onpattro^TM^) is probably the most impressive breakthrough in the drug discovery field in recent years. Patisiran is a double strand small interfering RNA (siRNA). Formulated as patisiran sodium, and with a molecular weight of 14,303.6 Da, this drug is encapsulated within a liposome nanoparticle for better delivery to the liver, where TTR is produced [14].

**Figure 2:**
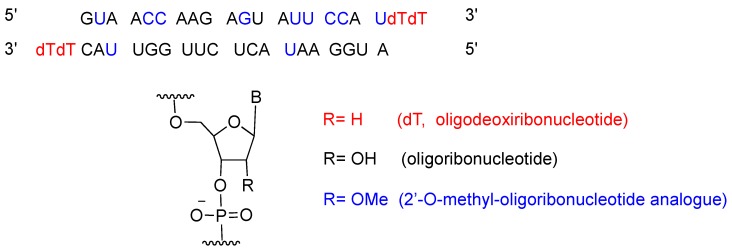
Patisiran sequence and chemical composition.

Patisiran is the first siRNA drug to use RNA interference (RNAi) to downregulate protein expression. The RNAi pathway was first described in 1998 by Fire and Mello [15], who were awarded the Nobel Prize in Medicine in 2006 for their work. Twenty years after the seminal publication, approval for patisiran was granted to Alnylam Pharmaceuticals, Inc. [16]. Patisiran had previously been granted Fast Track, Priority Review and Breakthrough Therapy, and Orphan Drug designations to facilitate its final approval. Figure 2 shows the patisiran sequence.

Patisiran is administered intravenously once every three weeks, and it has a demonstrated average knockdown of 87% and maximum of 96% of the TTR protein. Although, studies have shown that it is relatively well tolerated, it can cause some side effects, including tract infections, infusion-related reactions [13], and a decrease in vitamin A levels [13,17]. An important drawback of patisiran is the high cost of the treatment, which is calculated to be approximately $450,000 [18].

### 2.2. Inotersen (Tegsedi^TM^)

**Figure 3:**
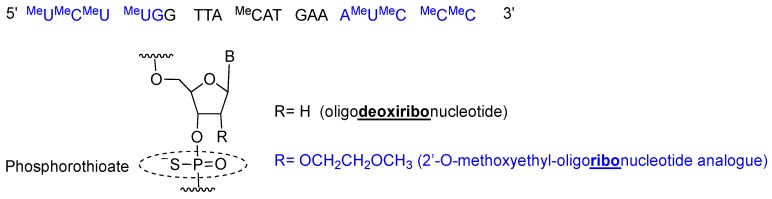
Inotersen sequence and chemical composition.

Inotersen is a single stranded 20-mer phosphorothioate antisense oligonucleotide with 10 central 2′-deoxyribonucleotides flanked by five 2′-O-methoxyethyl (MOE)-modified ribonucleotides at each of the 5′- and 3′-termini (5-10-5 gapmer structure) [19] (Figure 3). All pyrimidines (cytosine C and uracil U) are 5-methylated, thereby increasing affinity for the complementary chain. Once inotersen is hybridized to mRNA, the complex is degraded by RNaseH, reducing the total amount of TTR secreted by the liver [20].

Inotersen is formulated as sodium salt, with a molecular weight of 7600.8 Da [19]. Tegsedi was developed by Ionis Pharmaceuticals, Inc. and was approved by the FDA in October 2018 [21]. It was produced using solid-phase synthesis [19].

Inotersen is administered subcutaneously once a week, and it does not show major toxicity. However, it does cause some side effects, such as abnormally low levels of platelets (thrombocytopenia) and abnormal renal function (glomerulonephritis) [20]. Treatment with inotersen has a similar cost to that of patisiran [22].

### 2.3. Comments

Of the 59 drugs approved by the FDA in 2018, patisiran deserves to be named “Drug of the Year”. It is an oligonucleotide, where only six have been approved to date by the FDA, it shows a double strand, and requires a nanoformulation for its administration. From all points of view, patisiran could be considered a “tour de force” or in another context a “piece of art”. However, the main drawback is the investment needed for its development (some analysts are estimating this to be about US $ 2 billion) and the cost of treatment (US $ 450,000) [18]. Taking into account that its target is the same as that of inotersen, which has a similar treatment cost, these two drugs will compete for the same market, and are therefore unlikely to make a return on investment.

## 3. Lutathera^®^

**Figure 4:**
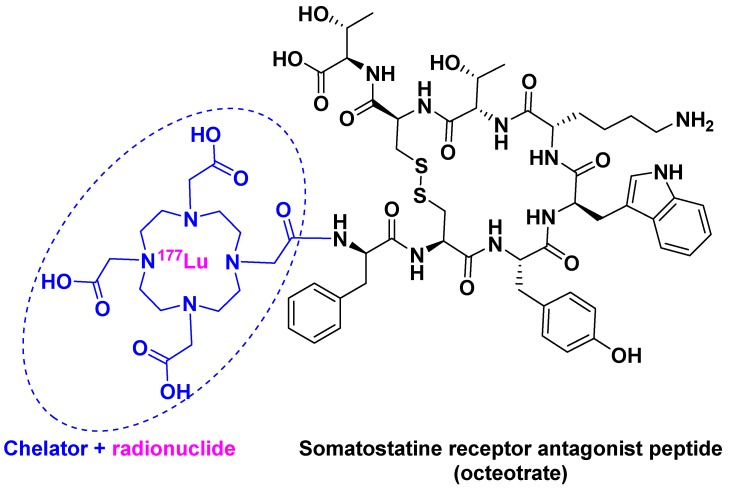
[^177^Lu]Lu-DOTA-TATE chemical structure.

[^177^Lu]Lu-DOTA-TATE ([[^177^Lu]Lu-DOTA^0^, Tyr^3^]-octreotate) belongs to an emerging treatment called Peptide Receptor Radionuclide Therapy (PRRT) [23]. PRRT involves the combination of a chelating agent holding the radionuclide and a peptide, which binds to receptors overexpressed by tumor cells. This mechanism facilitates localized treatment, and can therefore be considered a targeted therapy. [^177^Lu]Lu-DOTA-TATE is the first PRRT to have received FDA approval. It contains ^177^Lu as radionuclide and DOTA (also known as tetraxetan) as a chelator, the latter bound to [Tyr^3^]-octreotate (Figure 4). This peptide is a strong somatostatin receptor antagonist that is overexpressed in tumor cells [24]. The trivalent radiometal ^177^Lu has optimal β- and γ-emission characteristics, making it suitable to be used in theranostic radiopharmaceuticals as compared with other radionuclides: ^111^In for diagnostic and ^90^Y for therapy [25].

[^177^Lu]Lu-DOTA-TATE is administered intravenously, and has mild side effects, especially if kidney-protective agents are used during the therapy, in comparison to other PRRTs in development [23]. In addition, as a result of lower whole-body retention, [^177^Lu]Lu-DOTA-TATE has a lower risk for bone marrow toxicity [23].

[^177^Lu]Lu-DOTA-TATE is used for the treatment of gastroenteropancreatic neuroendocrine tumors (GEP-NETs). It was developed by Advanced Accelerator Applications USA, Inc. and then approved by the FDA in January 2018 [26].

In the case of PRRTs, as well as other similar radionuclide constructs used just for imaging, the main challenge is to widen the use of targeting peptides beyond somatostatin analogs. Research efforts should be channeled into more peptides from diverse families, with the aim of broadening the use of this kind of therapy.

## 4. Conclusions

The three TIDES approved by the FDA in 2018 can be considered unique. [^177^Lu]Lu-DOTA-TATE is the first drug of the PRRT family, patisiran is the first siRNA drug, and inotersen shows a distinctive gapmer structure. The acceptance of these drugs undoubtedly paves the way for the authorization of others with the same mode of action.

Finally, it is important to highlight that these and other drugs approved by the FDA in 2018 have a cost in the range of a six-digit figure, thus making them unaffordable for a large segment of the population.

## References

[1] Mullard A. (2017). 2016 FDA drug approvals. Nat. Rev. Drug Discov..

[2] Torre B.G., Albericio F. (2017). The pharmaceutical industry in 2016. An analysis of FDA drug approvals from a perspective of the molecule type. Molecules.

[3] Mullard A. (2018). 2017 FDA drug approvals. Nat. Rev. Drug Discov..

[4] De la Torre B.G., Albericio F. (2018). The pharmaceutical industry in 2017. An analysis of FDA drug approvals from the perspective of molecules. Molecules.

[5] Mullard A. (2019). 2018 FDA drug approvals. Nat. Rev. Drug Discov..

[6] De la Torre B.G., Albericio F. (2019). The pharmaceutical industry in 2018. An analysis of FDA drug approvals from the perspective of molecules. Molecules.

[7] Al Musaimi O., Al Shaer D., de la Torre B.G., Albericio F. (2018). 2017 FDA peptide harvest. Pharmaceuticals.

[8] Butler J.S., Chan A., Costelha S., Fishman S., Willoughby J.L., Borland T.D., Milstein S., Foster D.J., Goncalves P., Chen Q. (2016). Preclinical evaluation of rnai as a treatment for transthyretin-mediated amyloidosis. Amyloid.

[9] Rowczenio D.M., Noor I., Gillmore J.D., Lachmann H.J., Whelan C., Hawkins P.N., Obici L., Westermark P., Grateau G., Wechalekar A.D. (2014). Online registry for mutations in hereditary amyloidosis including nomenclature recommendations. Hum. Mutat..

[10] Chakradhar S. (2017). A protein puzzle. Nat. Med..

[11] Adams D., Suhr O.B., Dyck P.J., Litchy W.J., Leahy R.G., Chen J., Gollob J., Coelho T. (2017). Trial design and rationale for apollo, a phase 3, placebo-controlled study of patisiran in patients with hereditary attr amyloidosis with polyneuropathy. BMC Neurol..

[12] Vieira Simoes C.J., Lourenco de Almeida Z.C., Vasconcelos Dias de Pinho EMelo T.M., Pontes Meireles Ferreira de Brito R.M., Silva Costa D.C., Cabral Cardoso Lopes A.L. (2018). Bis-Furan Derivatives as Transthyretin (TTR) Stabilizers and Amyloid Inhibitors for the Treatment of Familial Amyloid Polyneuropathy (FAP). https://patents.google.com/patent/WO2016203402A1/ko.

[13] Hawkins P.N., Ando Y., Dispenzeri A., Gonzalez-Duarte A., Adams D., Suhr O.B. (2015). Evolving landscape in the management of transthyretin amyloidosis. Ann. Med..

[14] Yang J. (2019). Patisiran for the treatment of hereditary transthyretin-mediated amyloidosis. Exp. Rev. Clin. Pharmacol..

[15] Fire A., Xu S., Montgomery M.K., Kostas S.A., Driver S.E., Mello C.C. (1998). Potent and specific genetic interference by double-stranded rna in caenorhabditis elegans. Nature.

[16] FDA Approval Letter of Patisiran 2018. https://www.accessdata.fda.gov/drugsatfda_docs/appletter/2018/210922Orig1s000ltr.pdf.

[17] FDA Label of Patisiran 2018. https://www.accessdata.fda.gov/drugsatfda_docs/label/2018/210922s000lbl.pdf.

[18] Jarvis L.M. (2019). The new drugs of 2018. Chem. Eng. News.

[19] European Medicine Agency: Tegsedi Assessment Report 2018. https://www.ema.europa.eu/documents/assessment-report/tegsedi-epar-public-assessment-report_en.pdf.

[20] European Medicine Agency: Tegsedi Label. https://s3-us-west-2.amazonaws.com/drugbank/cite_this/attachments/files/000/001/938/original/tegsedi-epar-product-information_en.pdf?1539980614.

[21] FDA Approval Letter of Tegsedi 2018. https://www.accessdata.fda.gov/drugsatfda_docs/nda/2018/211172Orig1s000Approv.pdf.

[22] Fidler B. FDA Oks Akcea Rare Disease Drug, Setting Up Market Clash with Alnylam 2018. https://xconomy.com/boston/2018/10/05/fda-oks-akcea-rare-disease-drug-setting-up-market-clash-with-alnylam/.

[23] Kam B.L., Teunissen J.J., Krenning E.P., de Herder W.W., Khan S., van Vliet E.I., Kwekkeboom D.J. (2012). Lutetium-labelled peptides for therapy of neuroendocrine tumours. Eur. J. Nucl. Med. Mol. Imaging.

[24] Lutathera FDA Label 2018. https://www.accessdata.fda.gov/drugsatfda_docs/label/2018/208700s000lbl.pdf.

[25] Banerjee S., Pillai M.R., Knapp F.F. (2015). Lutetium-177 therapeutic radiopharmaceuticals: Linking chemistry, radiochemistry, and practical applications. Chem. Rev..

[26] FDA Approval Letter of Lutathera 2018. https://www.accessdata.fda.gov/drugsatfda_docs/appletter/2018/208700Orig1s000ltr.pdf.

